# Overexpression of *MdATG8i* improves water use efficiency in transgenic apple by modulating photosynthesis, osmotic balance, and autophagic activity under moderate water deficit

**DOI:** 10.1038/s41438-021-00521-2

**Published:** 2021-04-01

**Authors:** Xin Jia, Ke Mao, Ping Wang, Yu Wang, Xumei Jia, Liuqing Huo, Xun Sun, Runmin Che, Xiaoqing Gong, Fengwang Ma

**Affiliations:** 1 State Key Laboratory of Crop Stress Biology for Arid Areas/Shaanxi Key Laboratory of Apple, College of Horticulture, Northwest A&F University, 712100 Yangling, Shaanxi China; 2 Center of Pear Engineering Technology Research, State Key Laboratory of Crop Genetics and Germplasm Enhancement, College of Horticulture, Nanjing Agricultural University, 210095 Nanjing, China

**Keywords:** Photosynthesis, Drought, Stomata

## Abstract

Water deficit is one of the major limiting factors for apple (*Malus domestica*) production on the Loess Plateau, a major apple cultivation area in China. The identification of genes related to the regulation of water use efficiency (WUE) is a crucial aspect of crop breeding programs. As a conserved degradation and recycling mechanism in eukaryotes, autophagy has been reported to participate in various stress responses. However, the relationship between autophagy and WUE regulation has not been explored. We have shown that a crucial autophagy protein in apple, *MdATG8i*, plays a role in improving salt tolerance. Here, we explored its biological function in response to long-term moderate drought stress. The results showed that *MdATG8i-*overexpressing (*MdATG8i-*OE) apple plants exhibited higher WUE than wild-type (WT) plants under long-term moderate drought conditions. Plant WUE can be increased by improving photosynthetic efficiency. Osmoregulation plays a critical role in plant stress resistance and adaptation. Under long-term drought conditions, the photosynthetic capacity and accumulation of sugar and amino acids were higher in *MdATG8i-*OE plants than in WT plants. The increased photosynthetic capacity in the OE plants could be attributed to their ability to maintain optimal stomatal aperture, organized chloroplasts, and strong antioxidant activity. *MdATG8i* overexpression also promoted autophagic activity, which was likely related to the changes described above. In summary, our results demonstrate that *MdATG8i-*OE apple lines exhibited higher WUE than WT under long-term moderate drought conditions because they maintained robust photosynthesis, effective osmotic adjustment processes, and strong autophagic activity.

## Introduction

Drought stress is one of the most widespread environmental constraints and inhibits plant growth and production by affecting every aspect of the plant, especially plant turgor pressure, photosynthetic activity, organelle ultrastructure, metabolism, and hormones^1–3^. Due to global climate change and the increasingly limited available groundwater for agricultural use, drought stress has become a major threat to crop yields^4,5^. Hence, it is urgent to develop strategies and technologies to improve the water use efficiency (WUE) of crops. WUE can be defined in different ways; from the perspective of agricultural production, it is described as the amount of plant-produced biomass per unit of water consumed by plants. From a plant physiology viewpoint, WUE is defined as the ratio of the amount of CO_2_ fixed by photosynthesis to the amount of water vapor lost to the atmosphere^6^.

Photosynthesis plays a crucial role in plant growth because it produces carbohydrates and oxygen^7,8^. Greater photosynthetic capacity is one of the key factors involved in improving the WUE of crops without excessive yield penalties under long-term drought conditions. In recent studies, several genes related to photosynthesis have been manipulated to achieve the goal of increased productivity^9^. Under drought stress, the plant traits that maintain photosynthesis also increase plant growth and WUE^10^. However, photosynthetic activity is sensitive to water deficits because they result in a rapid decrease in stomatal aperture and damage to the photosynthetic machinery^11^. Photosystem II (PSII) is vulnerable to damage under various stresses, which results in reductions in electron transport and ATP synthesis^12,13^. In addition, pigment complexes and chloroplast structures can be destroyed by the excessive reactive oxygen species (ROS) generated under drought stress, resulting in diminished photosynthetic capacity^11^. Drought stress inhibits all aspects of the photosynthetic system and is ultimately harmful to plant growth.

Plants can alter various metabolic pathways to cope with drought stimuli, such as their carbohydrate and amino acid metabolism^14–17^. The soluble sugars and polyols accumulated under drought conditions can act as compatible osmolytes, ROS scavengers, energy sources, and signals^18–20^. Wei et al.^21^ demonstrated that tetraploid trifoliate orange was more drought tolerant than diploid trifoliate orange due to enhanced sugar accumulation. In addition, drought can induce the accumulation of amino acids, which contribute to maintaining cell turgor and removing excessive ROS^22^. For instance, proline can serve as an osmoprotectant and ROS scavenger, protecting plants from damage caused by drought and maintaining plant growth under long-term unfavorable environmental conditions^23^. The capacity to accumulate proline has been shown to be correlated with stress tolerance^24^. Furthermore, amino acids can also act as alternative energy sources at night, when plant starch resources are limited^25^.

Autophagy is a conserved eukaryotic mechanism for the degradation of unwanted or damaged proteins and organelles under stress conditions to maintain cell homeostasis^26^. Researchers have identified >40 autophagy-related genes (ATGs) in yeast^27^. Among them, 17 core ATGs are reported to participate in the formation of autophagosomes, namely, *ATG1-14*, *ATG16*, *ATG18*, and *ATG101*^28^. In particular, ATG8–PE (PE stands for phosphatidylethanolamine) and ATG5–ATG12, which are two ubiquitin-like protein conjugation systems, play a critical role in the expansion of the autophagosome membrane^28^. The ATG8 protein binds covalently to PE with the mediation of ATG3 and ATG7, and ATG12 binds to ATG5 with the mediation of ATG7 and ATG10^29^. In addition, the ATG5–ATG12 conjugation system is vital for the conjugation of ATG8 and PE. Over the years, homologs for these core ATGs have been identified in various plant species^27^.

In plants, autophagy occurs due to multiple environmental stresses, including drought and osmotic conditions^30,31^. *Arabidopsis atg5* and *atg7* mutants are hypersensitive to osmotic stress^30^. *Arabidopsis* RNAi‐*ATG18a* plants showed a greater reduction in growth than wild-type (WT) plants under drought and salt stresses^32^. Accordingly, the overexpression of *MdATG18a* improved tolerance to severe drought stress in transgenic apple^31^. The overexpression of *MdATG3s* isolated from apple in *Arabidopsis* increased their tolerance to osmotic stress^33^. In addition, the overexpression of *HsfA1a*, which regulates the expression of *ATG10* and *ATG18f* in tomatoes, resulted in higher autophagy activity that conferred drought tolerance^34^. These studies demonstrate that autophagy plays a positive role in improving plant tolerance to drought stress. However, the effect of autophagy on regulating WUE under long-term moderate drought conditions has not been explored.

The semiarid region of the Loess Plateau is one of the largest regions of apple production in China, accounting for nearly 25% of the total national apple production in 2016^35^. However, because of the sufficient light but uneven annual rainfall on the Loess Plateau, water deficit has become the primary limiting factor for apple production. Plants in this region face long-term moderate drought stress. The development of transgenic apple plants with high WUE and production is greatly needed. Previously, we cloned the apple ATG *MdATG8i* and demonstrated that it functions in apple autophagy in a conserved way^36^. *MdATG8i-*overexpressing (*MdATG8i*-OE) apple lines displayed improved salt tolerance and greater autophagic activity than WT plants^37^. In this study, we employed *MdATG8i-*OE apple plants to investigate the function of this gene under long-term moderate drought conditions. We found it particularly interesting that, compared with WT plants, *MdATG8i-*OE apple plants exhibited higher WUE with minor growth penalties under long-term moderate drought stress, possibly because the OE plants exhibited higher autophagic activity, greater photosynthetic capacity, and better osmotic adjustment. This study provides a promising approach for improving WUE with minimized growth costs in apple under long-term drought stress.

## Results

### Overexpression of *MdATG8i* improves growth and WUE in apple under long-term moderate drought stress

**Fig. 1:**
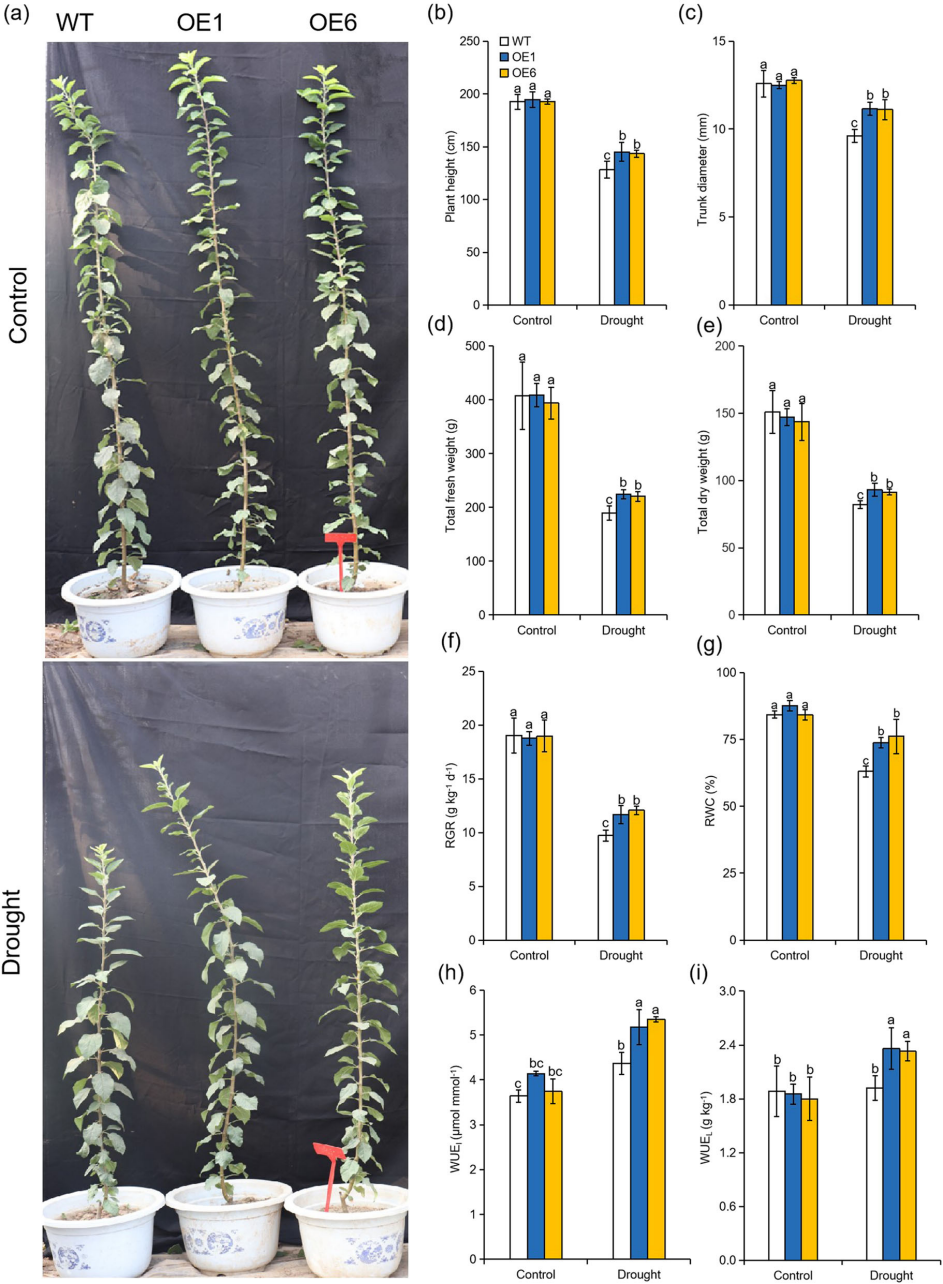
Overexpression of *MdATG8i* promotes growth and water use efficiency (WUE) in apple plants under long-term moderate drought conditions. Water was withheld to maintain the soil at 45–50% field capacity from June 20, 2018 to September 8, 2018. **a** Growth phenotypes of WT plants and *MdATG8i*-overexpressing apple lines after 80 days of growth under normal or moderate drought stress conditions. Comparisons of plant height (**b**), trunk diameter (**c**), total fresh weight (**d**), total dry weight (**e**), relative growth rate (RGR; **f**), relative water content (RWC; **g**), instantaneous water use efficiency (WUE_I_; **h**), and long-term water use efficiency (WUE_L_; **i**) between WT and transgenic plants under 80 days of normal or moderate drought stress conditions. The data are shown as the means of six replicates with SDs. Values with different letters differed significantly at *P* < 0.05 according to Tukey’s multiple range test.

*MdATG8i* transcription can be induced by sustained moderate drought stress in GL-3 plants (Fig. S1), indicating the potential involvement of *MdATG8i* in drought tolerance. To analyze the biological function of *MdATG8i* under long-term moderate drought stress, two previously obtained transgenic lines of *MdATG8i-*OE apple were used for further treatment^37^. We investigated the phenotypes of the WT plants and *MdATG8i* overexpression lines after 80 days of long-term drought treatment (water withheld until 45–50% soil moisture content was reached). The growth phenotypes of the WT and OE plants were not markedly different at the beginning of the long-term experiment or after 80 days normal watering (Fig. S2 and Fig. 1a). After 80 days of the moderate drought treatment, the growth phenotypes were significantly affected in terms of the plant height, trunk diameter, total fresh weight (TFW), total dry weight (TDW), and relative growth rate (RGR). However, the growth of the OE plants was less affected than that of the WT plants under long-term moderate drought conditions. Under sustained moderate drought stress, the transgenic plants had higher plant height and trunk diameter than the WT plants (Fig. 1a–c). Compared with the WT plants, the *MdATG8i-*OE apple lines accumulated more biomass and maintained a higher RGR after 80 days of the moderate drought treatment (Fig. 1d–f). Additionally, the water deficit treatment for 80 days led to decreased RWC in all genotypes, but the OE lines had significantly higher RWC values than the WT plants (Fig. 1g). High WUE is crucial for plants in adapting to long-term water deficit^38^. As shown in Fig. 1h, after 80 days of moderate drought, the instantaneous WUE (WUE_I_) was higher in the *MdATG8i-*OE plants than in the WT plants. Furthermore, after 80 days of drought treatment, the long-term WUE (WUE_L_) values of OE1 and OE6 were 22.7 and 21.2%, respectively, higher than that of WT (Fig. 1i). These data indicate that *MdATG8i* positively regulated the accumulation of biomass and WUE in apple under long-term drought stress.

### Overexpression of *MdATG8i* enhances photosynthetic capacity in apple under long-term moderate drought stress

**Fig. 2:**
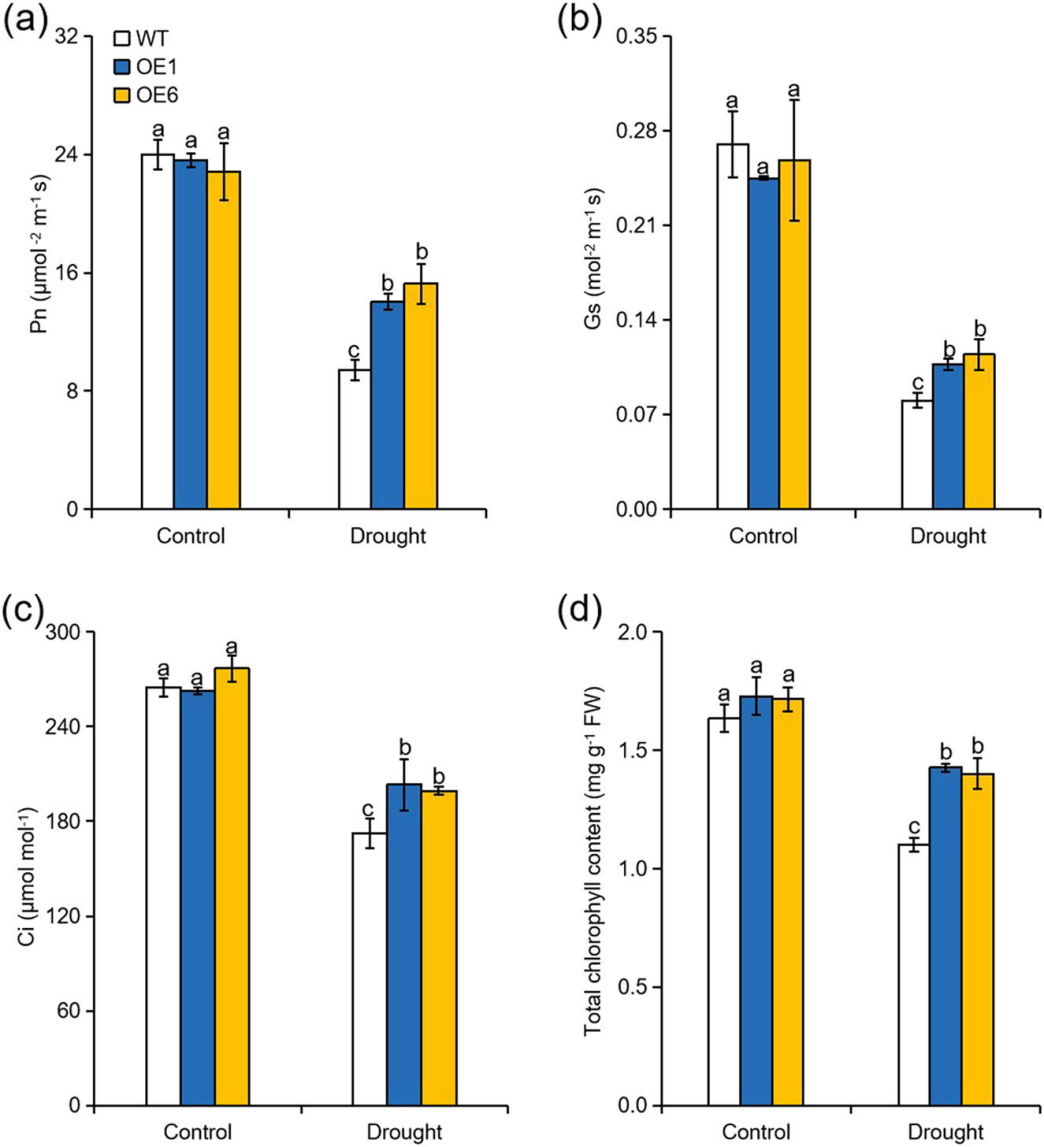
Effect of *MdATG8i* overexpression in apple on the photosynthetic parameters under normal or moderate drought stress conditions. Effect of *MdATG8i* overexpression in apple on the photosynthetic capacity under normal or moderate drought stress conditions. Comparisons of photosynthetic rate (Pn; **a**), stomatal conductance (Gs; **b**), intercellular CO_2_ concentration (Ci; **c**), and chlorophyll concentration (**d**) between *MdATG8i*-overexpressing apple lines and WT plants under 80 days of normal or moderate drought conditions. The data are shown as the means of five replicates with SDs. Values with different letters differed significantly at *P* < 0.05 according to Tukey’s multiple range test.

Since there was a difference in growth between the WT and *MdATG8i-*OE plants after 80 days of drought treatment, we investigated whether there was also a difference in their photosynthetic capacity. We measured the gas exchange parameters and chlorophyll concentrations of all genotypes in the long-term moderate drought treatment. No significant differences in photosynthetic rate (Pn), stomatal conductance (Gs), intercellular CO_2_ concentration (Ci), or chlorophyll concentration were observed between the WT and OE plants under normal conditions. These four parameters decreased sharply under sustained moderate drought stress, but the decrease was less severe in the *MdATG8i-*OE plants than in the WT plants (Fig. 2a–d). After 80 days of the moderate drought treatment, the Pn values of both OE lines were approximately 1.7 times higher than those of the WT plants (Fig. 2a). The Gs, Ci, and chlorophyll concentrations exhibited similar trends (Fig. 2b–d). These results suggest that the *MdATG8i* overexpression lines exhibited higher photosynthetic capacity than WT under the long-term drought treatment.

### Overexpression of *MdATG8i* alters stomatal parameters in apple under long-term moderate drought stress

**Fig. 3:**
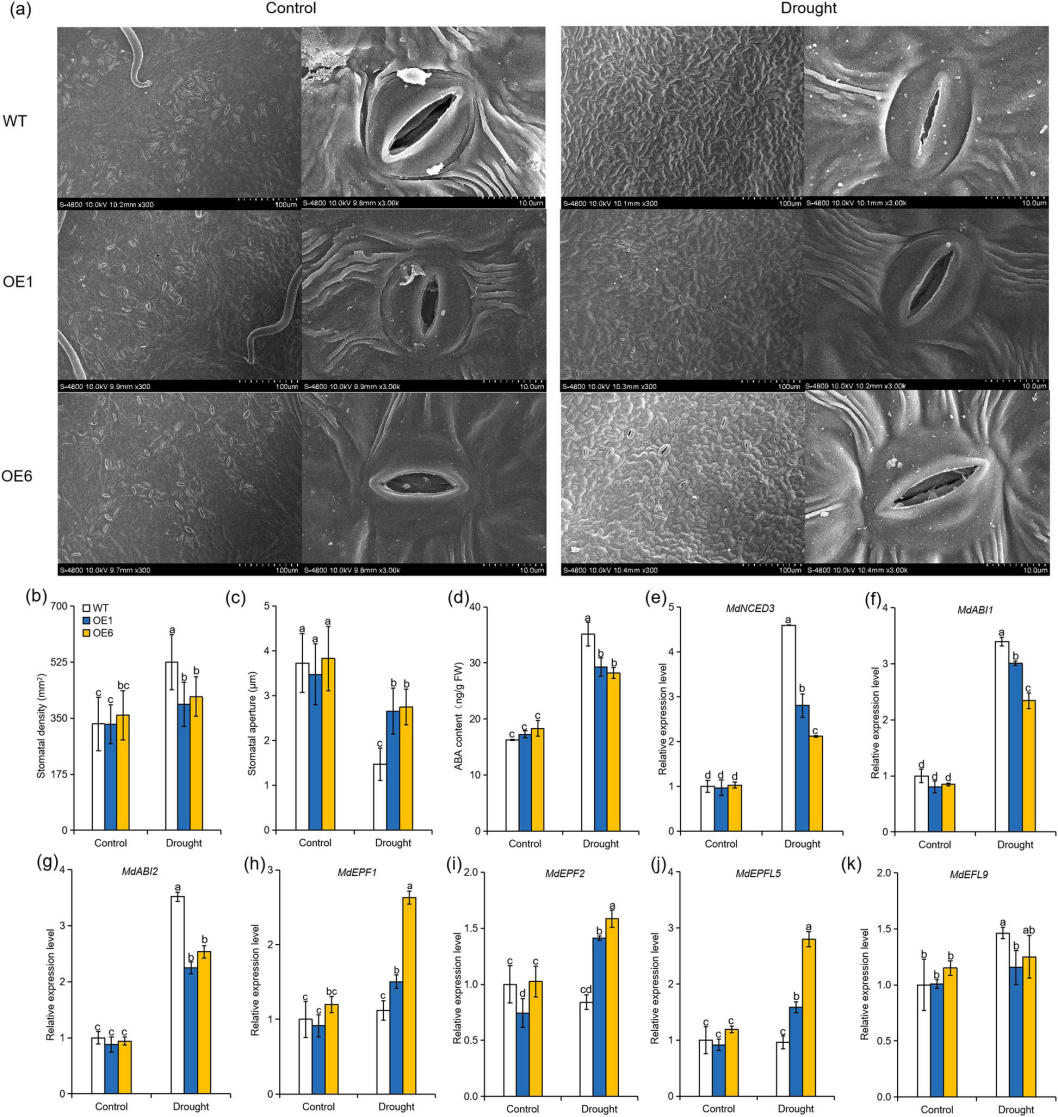
Stomatal characteristics and ABA content in *MdATG8i*-overexpressing apple lines and WT plants under normal or moderate drought stress conditions. **a** Representative stomatal images observed by scanning electron microscopy (SEM). Comparisons of stomatal density (**b**), stomatal aperture (**c**), and ABA content (**d**) between the WT and transgenic plants. Gene expression analysis of *MdNCED3* (**e**), *MdABI* (**f**), *MdABI2* (**g**), *MdEPF1* (**h**), *MdEPF2* (**i**), *MdEPFL5* (**j**), and *MdEPFL9* (**k**) in the WT and OE lines under 80 days of normal or moderate drought stress conditions. The stomatal characteristics data are presented as means ± SDs (*n* > 30). The ABA content and gene expression data are the means of three replicates with SD. Values with different letters differed significantly at *P* < 0.05 according to Tukey’s multiple range test.

Drought stress can affect stomatal parameters, which are closely related to the photosynthetic activity and water-holding capacity of plants^39^. Under well-watered growth conditions, there was no difference in stomatal characteristics between the genotypes. The stomatal density was increased in all genotypes after 80 days of drought stress, but the OE plants maintained lower stomatal density than the WT plants (Fig. 3a, b). Meanwhile, the stomatal apertures in the *MdATG8i-*OE plants were less affected by long-term drought stress than those in the WT plants (Fig. 3c). The ABA level consistently increased more in the WT plants than in the *MdATG8i-*OE plants in the long-term drought treatment (Fig. 3d). The transcription levels of a key ABA biosynthesis gene and two ABA-responsive genes (*MdNCED3*, *MdABI1*, and *MdABI2*, respectively) were induced more in the WT plants than in the OE plants under drought stress (Fig. 3e–g). In *Arabidopsis*, epidermal patterning factor (EPF) and EPF-LIKE (EPFL) encode a secreted peptide family (*EPF1*, *EPF2*, and *EPFL1-9*) that plays a vital role in the stomatal development process^40^. It has been reported that *EPF1* and *EPF2* negatively regulate stomatal development, whereas *EPFL9* regulates stomatal development positively^41–43^. Jiang et al.^42^ demonstrated that the ectopic expression of *MdEPF2* in tomato led to a reduction in stomatal density in transgenic plants. To investigate the reason for the difference in stomatal development between genotypes under long-term drought conditions, we examined the expression level of several *MdEPF* family genes, i.e., *MdEPF1*, *MdEPF2*, *MdEPFL5*, and *MdEPFL9*. As shown in Fig. 3h–j, the transcription levels of *MdEPF1*, *MdEPF2*, and *MdEPFL5* were more significantly induced by drought stress in the *MdATG8i-*OE plants than in the WT plants. In contrast, the transcription level of *MdEPFL9* was higher in the WT than in the OE plants under drought conditions (Fig. 3k). These data indicate that the overexpression of *MdATG8i* could influence stomatal behavior and development under sustained moderate drought conditions.

### Overexpression of *MdATG8i* stimulates ROS scavenging in apple plants under long-term moderate drought stress

**Fig. 4:**
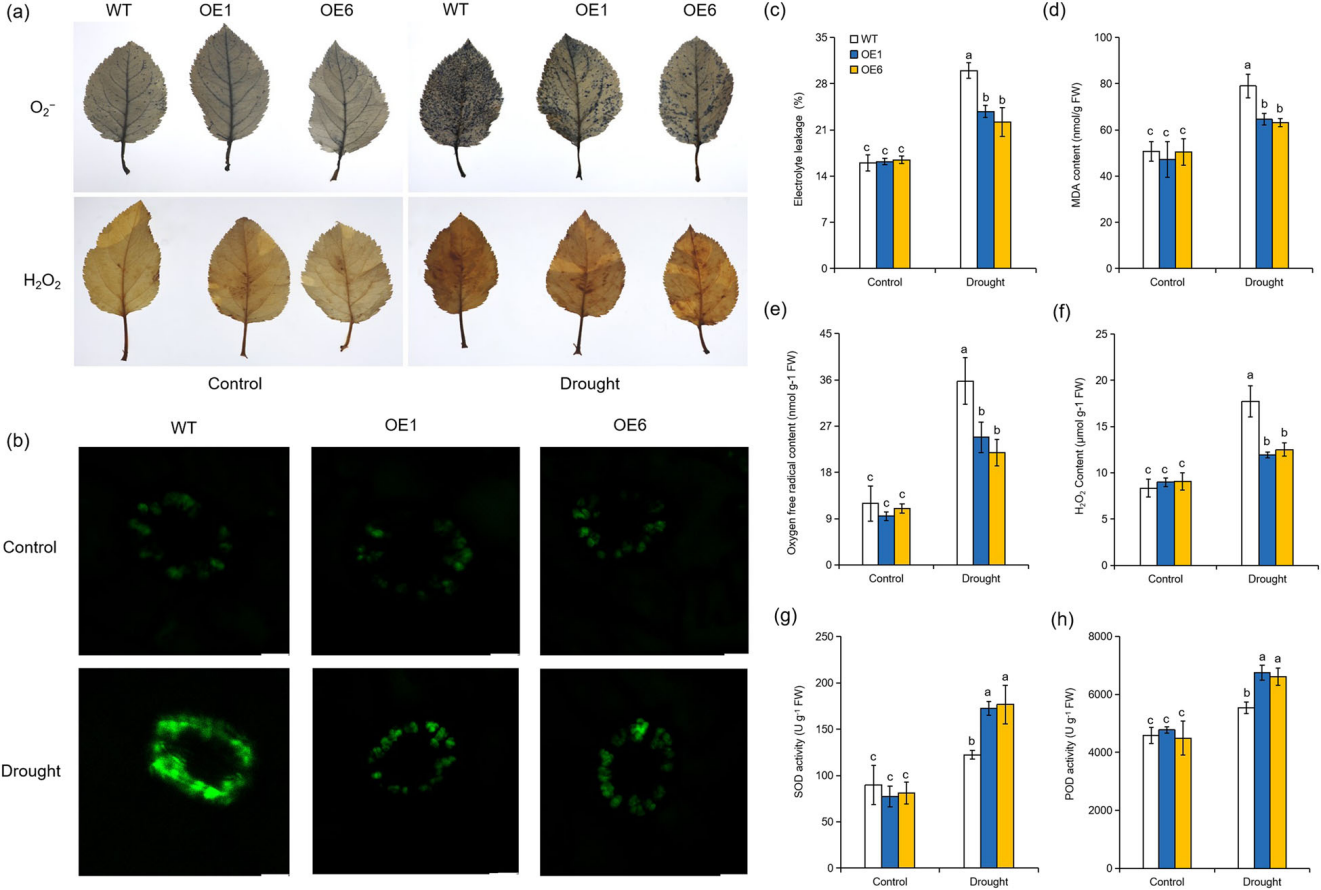
*MdATG8i* overexpression improves ROS scavenging ability in response to long-term moderate drought conditions. **a** Representative images of leaves showing the accumulation of O_2_^−^ and H_2_O_2_ as revealed by staining with NBT and DAB, respectively, in response to drought stress. **b** Fluorescence detection of ROS accumulation in guard cells of *MdATG8i*-overexpressing apple lines and WT plants using H_2_DCFDA under normal or drought stress conditions. Comparisons of electrolyte leakage (c) and MDA concentrations (**d**) between WT and transgenic plants. Quantitative measurements of the O_2_^−^ (**e**) and H_2_O_2_ concentrations (**f**) in apple leaves after 80 days of normal or moderate drought conditions. Activities of superoxide dismutase (SOD; **g**) and peroxidase (POD; **h**) after 80 days of normal or moderate drought conditions. The data are the means of three replicates with SDs. Values with different letters differed significantly at *P* < 0.05 according to Tukey’s multiple range test.

Under normal growth conditions, electrolyte leakage and malondialdehyde (MDA) concentrations did not differ substantially among the *MdATG8i-*OE apple lines and the WT plants. Although the 80-day water deficit led to increased electrolyte leakage and MDA concentrations in apple, the OE plants maintained lower electrolyte leakage values and MDA concentrations than the WT plants (Fig. 4c, d). Drought can induce excessive ROS accumulation, which subsequently damages various cell components. After 80 days of the moderate drought treatment, we stained the leaves with 3,3’-diaminobenzidine (DAB) for hydrogen peroxide (H_2_O_2_) detection and nitroblue tetrazolium (NBT) for oxygen free radical (O_2_^−^) detection. As shown in Fig. 4a, sustained moderate drought induced ROS accumulation in all genotypes, but more brown areas and blue spots were observed in the leaves of the WT plants than in the leaves of the *MdATG8i-*OE apple lines. We also measured the ROS levels in guard cells with 2ʹ,7ʹ-dichlorofluorescin diacetate (H_2_DCFDA), an oxidation-sensitive fluorescence probe. The intracellular ROS levels under drought stress were higher in the WT plants than in the OE lines (Fig. 4b). These observations were consistent with the quantitative measurements of H_2_O_2_ and O_2_^−^, showing that the OE plants accumulated less ROS than the WT plants under sustained moderate drought conditions (Fig. 4e, f). Furthermore, the activities of superoxide dismutase (SOD) and peroxidase (POD) significantly increased under drought conditions, and a larger increase occurred in the OE lines than in the WT plants (Fig. 4g, h). These results suggest that the *MdATG8i-*OE lines maintained lower ROS accumulation than WT under drought conditions.

### Overexpression of *MdATG8i* improves energy conversion efficiency in apple under long-term moderate drought stress

**Fig. 5:**
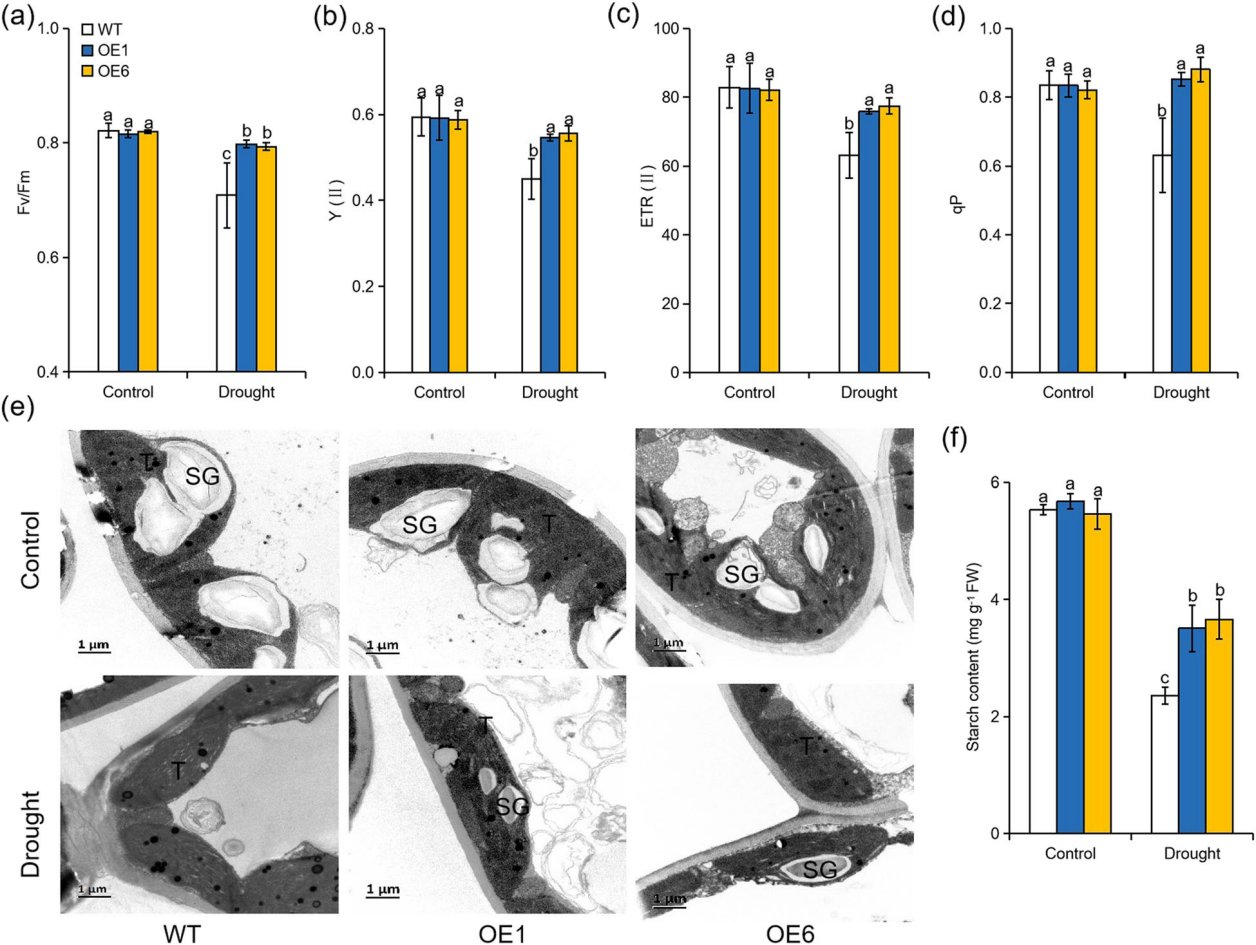
Comparisons of chlorophyll fluorescence parameters and chloroplast structures between *MdATG8i*-overexpressing and WT plants under 80 days of normal or drought conditions. **a** Maximum quantum yield of PSII (Fv/Fm). **b** Effective quantum yield of PSII [Y(II)]. **c** Electron transport rate of PSII [ETR (II)]. **d** Photochemical quenching (qP). **e** Transmission electron microscopic (TEM) images of chloroplasts in leaves from *MdATG8i*-overexpressing apple lines and WT plants. T thylakoid, SG starch granules. Scale bars = 1 μm. **f** Starch concentrations under normal or drought conditions. The data are the means of three replicates with SDs. Values with the different letters differed significantly at *P* < 0.05 according to Tukey’s multiple range test.

The measurement of chlorophyll fluorescence is widely used for obtaining information on the light energy conversion efficiency of PSII. Under well-watered conditions, no significant differences in Fv/Fm, Y(II), ETR(II), and qP were observed among all genotypes (Fig. 5a–d). After 80 days of the moderate drought treatment, the values of Fv/Fm, Y(II), ETR(II), and qP, which reflect energy conversion in PSII, decreased due to the injury caused by sustained moderate drought. However, the values were significantly lower in the WT plants than in the *MdATG8i-*OE plants (Fig. 5a–d). Here, we also observed changes in the chloroplast structures of the WT and *MdATG8i-*OE plants under the sustained moderate drought treatment. Under normal conditions, the chloroplasts were well organized, and the grana were stacked closely in the chloroplasts of the leaves of all genotypes (Fig. 5e). However, the WT plants had a much reduced thylakoid membrane network and less grana stacking in the chloroplasts than the OE lines under long-term drought stress. Additionally, there were more starch granules in the chloroplasts of the OE lines under drought stress (Fig. 5e). Accordingly, the starch content in the mature leaves was notably higher in the *MdATG8i-*OE plants than in the WT plants under the sustained moderate drought treatment (Fig. 5f). Thus, the overexpression of *MdATG8i* appeared to improve WUE by mitigating damage to the photosynthetic apparatus under long-term drought stress.

### Overexpression of *MdATG8i* improves the accumulation of soluble sugars and amino acids in apple under long-term moderate drought stress

**Fig. 6:**
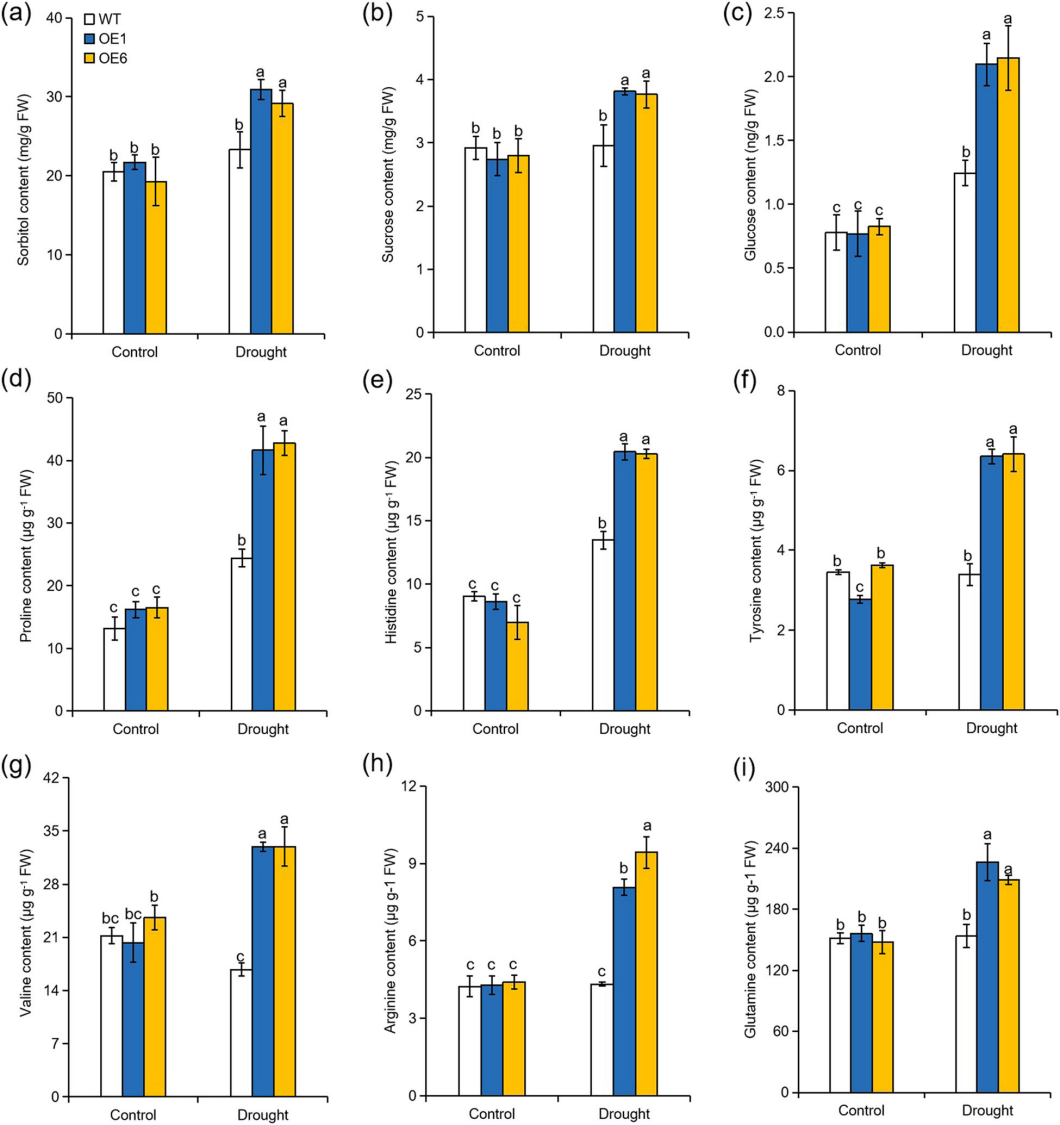
Accumulations of soluble sugars and amino acids in the leaves of *MdATG8i*-overexpressing apple lines and WT plants after 80 days of normal or drought stress conditions. Comparisons of sorbitol (**a**), sucrose (**b**), glucose (**c**), proline (**d**), histidine (**e**), tyrosine (**f**), valine (g), arginine (**h**), and glutamine (**i**) contents between WT and *MdATG8i*-overexpressing apple plants. The data are shown as the means of three replicates with SDs. Values with different letters differed significantly at *P* < 0.05 according to Tukey’s multiple range test.

Soluble sugars, which can act as compatible solutes, energy sources, and signals, can be induced by various stresses^44^. In our study, the concentrations of sorbitol, sucrose, and glucose were significantly elevated by drought stress, and their levels were significantly higher in the *MdATG8i-*OE plants than in the WT plants (Fig. 6a–c). The transcription levels of the genes encoding the glucose sensor hexokinase (*MdHXK1*), neutral invertase (*MdNINV1/2*), cell wall invertase (*MdCWINV1/2*), and sucrose phosphate synthase (MdSPS6) were elevated more in the *MdATG8i-*OE plants than in the WT plants in response to the sustained drought stress (Fig. S3).

To investigate whether *MdATG8i-*mediated drought tolerance was also involved in amino acid metabolism, we determined the levels of 18 amino acids in all genotypes from each treatment. The results are listed in Fig. S4 and Fig. 6d–i. In response to sustained moderate drought stress, the transgenic lines accumulated more of almost all the measured amino acids than the WT plants. However, the concentrations of several amino acids were notably different between the WT and *MdATG8i-*OE plants under long-term drought conditions. For example, the levels of proline in the OE plants were approximately 1.75-fold those in the WT plants after 80 days of drought treatment (Fig. 6d). In addition, the levels of glutamine, histidine, arginine, tyrosine, and valine were approximately 1.4, 1.5, 2.0, 1.88, and 1.97 times higher, respectively, in the *MdATG8i-*OE lines than in WT under drought conditions (Fig. 6e–i).

Thus, overexpression of *MdATG8i* appeared to improve adaptation to drought stress in apple by elevating the accumulation of carbohydrates and amino acids.

### Overexpression of *MdATG8i* enhances the autophagic activity under long-term moderate drought stress in apple

**Fig. 7:**
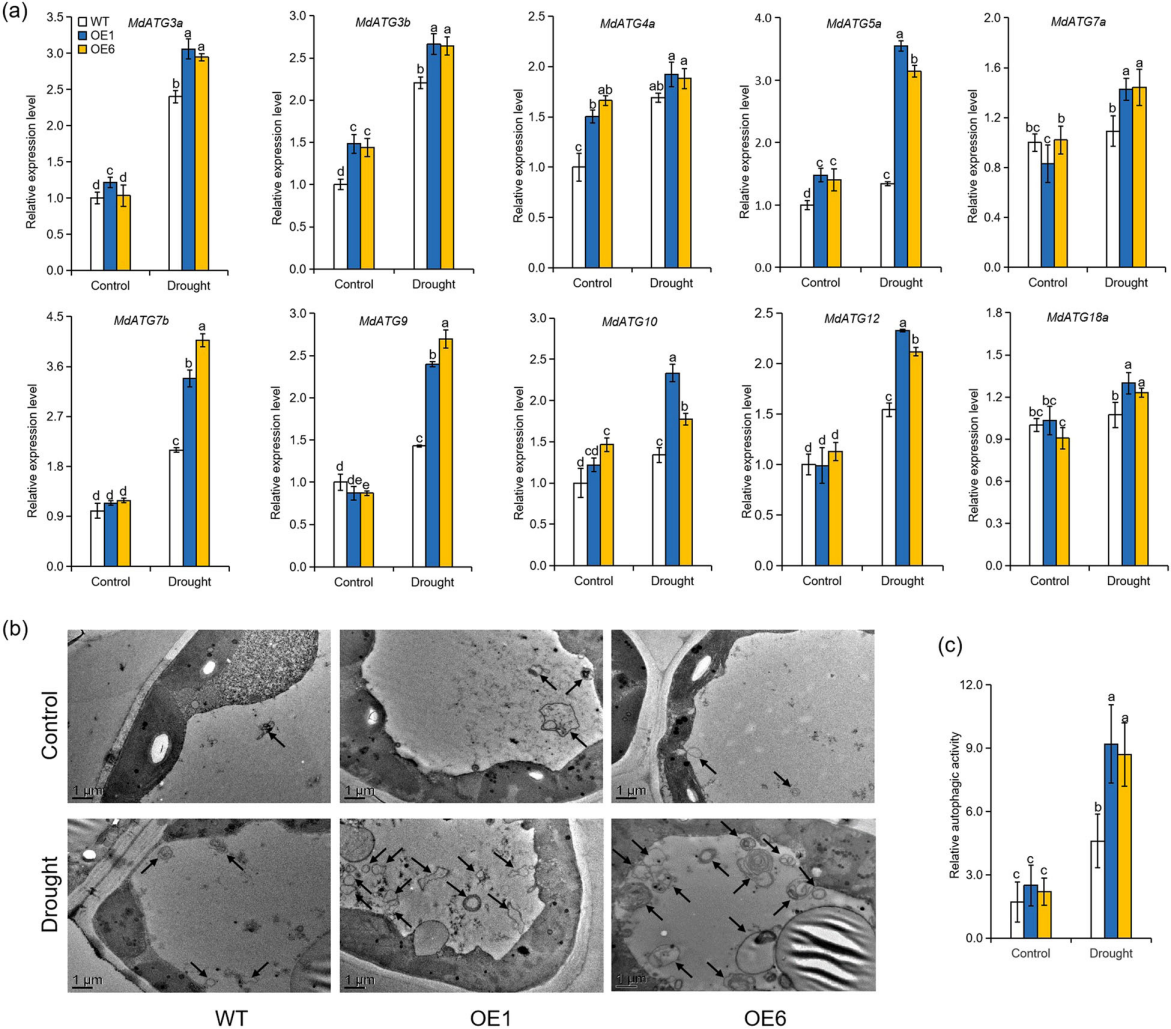
Transcription levels of apple autophagy-related genes and autophagosomes accumulation in the leaves of *MdATG8i*-overexpressing apple lines and WT plants after 80 days of a moderate drought treatment. **a** Expression levels of apple autophagy-related genes in the leaves of WT and *MdATG8i*-overexpressing apple plants under moderate drought conditions. **b** Transmission electron microscopic (TEM) images of the autophagic structures in the leaves of WT and *MdATG8i*-overexpressing apple plants. The autophagic structures are shown by black arrows. Scale bars: 1 μm. **c** Relative autophagic activity represented as a comparison of the activities of the WT or transgenic plants shown in **b**. The results are represented as the means ± SDs. Values with different letters differed significantly at *P* < 0.05 according to Tukey’s multiple range test.

To investigate whether *MdATG8i* overexpression was related to autophagy under moderate drought conditions, we first determined the transcription levels of several core *MdATG*s that are ATG homologs in *Malus domestica*. Under well-watered conditions, the transcription levels of *MdATG3a*, *MdATG7a*, *MdATG7b*, *MdATG9*, *MdATG10*, *MdATG12*, and *MdATG18a* were similar in all genotypes, whereas the transcription levels of *MdATG3b*, *MdATG4a*, and *MdATG5a* were higher in the *MdATG8i-*OE plants than in the WT plants (Fig. 7a). As shown in Fig. 7a, the transcription of almost all tested genes could be induced by sustained moderate drought treatment, and this effect was more pronounced in the *MdATG8i-*OE plants than in the WT plants. To confirm these results, we measured the formation of autophagosomes under long-term drought stress using transmission electron microscopy. Under well-watered conditions, very few autophagic structures were observed in all genotypes. After 80 days of water deficit, the number of autophagic structures in all genotypes increased significantly, and there were nearly twice as many autophagic structures in the *MdATG8i-*OE plants as in the WT plants (Fig. 7b, c). These results show that the overexpression of *MdATG8i* contributed to increasing autophagic activity in apple plants exposed to long-term moderate drought stress.

## Discussion

Due to global climate change, water deficit has become a serious problem that limits plant growth and crop yield worldwide^45^. It is important to develop traits in plants that improve WUE without increasing production costs^46^. Autophagy has been previously reported to play a significant role in improving plant tolerance to various types of stress^26,34,47–50^. Our group also conducted several studies related to the potential effects of ATGs on abiotic stress tolerance in apple. For example, we identified *MdATG3a* and *MdATG3b* and found that their ectopic expression improved osmotic stress tolerance in *Arabidopsis*^33^. The overexpression of *MdATG18a* enhanced drought tolerance in apple by modifying the antioxidant system and increasing autophagic activity^31^. A recent study showed that *MdATG8i* regulated photosynthesis and the accumulation of polyamines, thus enhancing salt stress tolerance in apple^37^. Instead of looking at the effect of autophagy on tolerance to short-term, severe stresses, as our past studies have done, this work examines the role of autophagy in modulating long-term, adaptive response under moderate drought. In both the short-term stress response and long-term adaptation in apple plants, the ATG was found to be related to changes in antioxidant capacity and upregulated autophagy. Notably, this study demonstrated that *MdATG8i-*OE-mediated autophagy functioned positively in the adaptive response of apple to long-term drought by maintaining plant growth and improving WUE, which may have been achieved by exerting influence on some adaptive strategies (i.e., maintaining the optimal stomatal aperture, developing organized chloroplasts, and making osmotic adjustments). In summary, this research, together with previous studies, leads to the conclusion that autophagy is a key factor regulating both the stress response and the long-term adaptive response in apple.

Photosynthesis is a key physiological process that directly affects plant production and WUE^10,51^. Enhancing the photosynthetic ability of plants with slight changes in Gs is considered an ideal approach to improving both yield and WUE^10^. Zhou et al.^38^ demonstrated that photosynthesis regulation contributed significantly to higher WUE in apple under drought stress. Autophagy may have a positive effect on the photosynthetic capacity of plants. Chloroplast proteins that are involved in photosynthesis are less abundant in autophagy-defective mutants under nutrient-deficient conditions^52,53^. In this study, drought stress decreased the photosynthetic efficiency of all genotypes. However, we found that the levels of photosynthetic efficiency in the *MdATG8i-*OE plants were higher than those in the WT plants under moderate drought conditions. Therefore, the overexpression of *MdATG8i* appeared to modulate WUE by improving the photosynthetic system in apple under long-term moderate drought conditions.

Drought stress affects the photosynthetic efficiency of plants primarily through stomatal closure^54^. Some studies have suggested that maintaining a certain degree of stomatal opening benefits plant growth under long-term moderate drought stress^55,56^. Wang et al.^56^ demonstrated that the apple cultivar “Qinguan,” which has high WUE, exhibited greater stomatal aperture than the cultivar “Honeycrisp,” which has low WUE, under long-term moderate drought conditions. It is more conducive to plant growth and WUE to maintain a certain degree of stomatal opening in a long-term but relatively mild drought. The phytohormone ABA plays a crucial role in regulating stomatal aperture under drought conditions^57^. It has previously been reported that ABA contents were elevated in the leaves of the autophagy-defective mutant *atg12* even under normal conditions^52^. Accordingly, we found that the Gs and stomatal apertures in the *MdATG8i-*OE plants were both larger than those in the WT plants under long-term drought stress; this may have been due to the low ABA level in the OE plants. In addition, autophagy has been reported to help keep the stomata open by regulating ROS homeostasis in guard cells^58^. Here, the low intracellular ROS levels in the guard cells of the OE plants might have been responsible for the stomatal phenotype under drought stress. The optimized stomatal apertures in the OE plants resulted in improved photosynthetic capacity, which eventually contributed to better growth performance and greater WUE in the transgenic lines than in WT.

In addition to stomatal limitations, nonstomatal limiting factors that damage the photosynthetic apparatus under drought stress also affect the photosynthetic capacity of plants^11,55^. PSII is the part of the photosynthetic apparatus and is the most vulnerable target of multiple stresses^13,59^. In this study, the *MdATG8i-*OE plants had higher light energy conversion efficiency than the WT plants under long-term drought conditions, which demonstrated the protective effect of autophagy on PSII. It has been previously reported that autophagy contributes to degrading damaged chloroplasts to control the quality of cell chloroplasts when plants are subjected to ultraviolet B light or heat stress^49,60^. Consistently, we also found that the transgenic lines showed less damage to their chloroplasts than the WT plants after 80 days of the drought treatment. In this context, *MdATG8i*-mediated autophagy may play a role in improving photosynthesis by protecting the photosynthetic apparatus in apple under long-term drought conditions. Furthermore, the toxic ROS generated under drought stress can destroy the photosynthetic apparatus^11^. The improved photosynthesis in the OE plants might in part be the result of better antioxidant system functioning under long-term drought conditions. Moreover, the milder chloroplast damage and more active PSII in the OE plants also led to the generation of fewer toxic ROS during the drought treatment.

Under water-limited conditions, plants accumulate various compatible solutes to make osmotic adjustments, including soluble sugars and amino acids^61–64^. Previous works have demonstrated that osmotic adjustment is an important drought adaptation strategy that supports plant growth and development^65^. It has been previously reported that amino acid and sugar metabolism is substantially modified in autophagy mutants^66,67^. The autophagic process yields sugars, amino acids, and fatty acids that can be utilized by plants as anabolic substrates or for energy production^68,69^. Sun et al.^50^ demonstrated that *MdATG18a* overexpression promoted the accumulation of carbohydrates in apple under N-depletion conditions. In our research, the OE plants accumulated more soluble sugars and amino acids than the WT plants under long-term moderate drought, leading to better osmotic adjustment and stronger protection of the photosynthetic apparatus. The favorable osmotic adjustment in OE plants contributed to mitigating the negative impacts of drought on plant growth. In addition, the accumulated starch, soluble sugars, and amino acids in the OE plants could act as energy sources under drought conditions and might have been partly responsible for the improved growth and WUE^25,70^. Due to the active metabolism of sugar and amino acids, the *MdATG8i-*OE plants had higher WUE and were more adaptive to water deficit stress than the WT plants. However, the metabolism of sugar and amino acids can be influenced by multiple factors, and the specific mechanism by which autophagy affects metabolism under drought conditions requires further exploration.

Usually, plants undergo growth–defense trade-offs when subjected to harsh environmental conditions, and optimal growth traits are achieved at the cost of compromised stress tolerance^71^. Previous studies have indicated that manipulation of autophagy has an effect on various aspects of plant fitness, including vegetative growth and stress tolerance^58,72^. Minina et al.^73^ found that enhanced growth in *ATG5*- or *ATG7*-OE *Arabidopsis thaliana* was accompanied by improved tolerance to oxidative stress. Similarly, in our research, the enhanced autophagic activity in *MdATG8i-*OE plants resulted in better growth under long-term drought conditions without sacrificing stress tolerance, ultimately leading to improved WUE and drought adaptability. Our results demonstrate that a desirable agronomic trait, high plant WUE with minimal costs to growth, could be developed by modulating autophagy.

## Conclusions

**Fig. 8:**
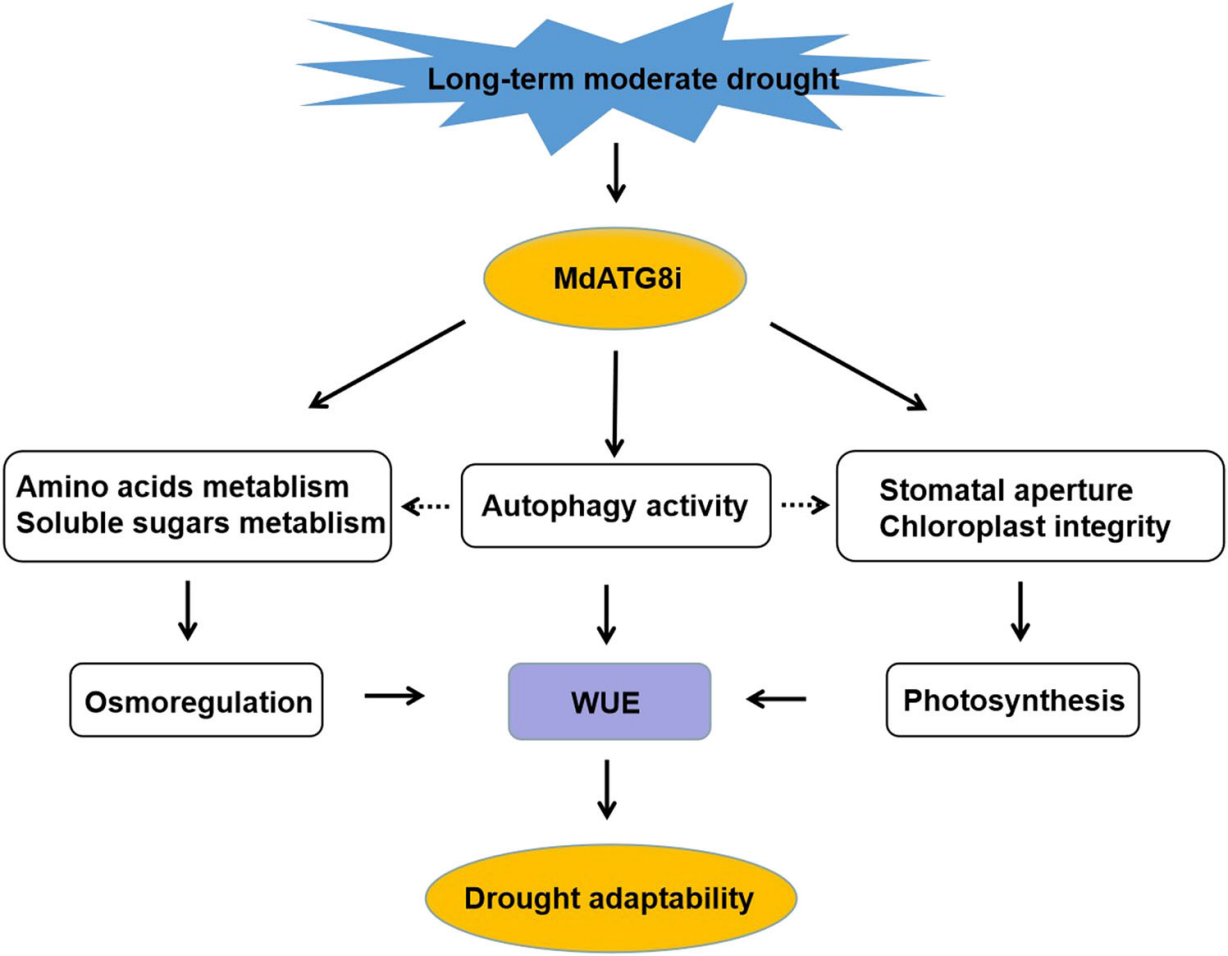
A proposed model showing the regulatory function of MdATG8i in response to long-term moderate drought stress in apple. Under long-term moderate drought conditions, *MdATG8i* overexpression contributes to maintaining optimal stomatal aperture and organized chloroplasts, resulting in greater photosynthetic capacity and thus higher WUE. Moreover, increased expression of *MdATG8i* also modulates the accumulation of sugar and amino acids in response to long-term drought stress, resulting in better osmotic adjustment as well as higher WUE and drought adaptability. Most importantly, *MdATG8i* overexpression promotes autophagic activity, which was likely related to the above changes, eventually resulting in improved WUE and drought adaptability.

In summary, we analyzed the effect of *MdATG8i* on WUE regulation and plant adaption to drought in apple (Fig. 8). Our results demonstrated that *MdATG8i-*OE plants exhibited high WUE and drought adaptability with minor biomass penalties under long-term drought conditions. This response could be attributed to the enhanced autophagic activity, greater photosynthetic capacity, and improved osmotic adjustment in the transgenic plants. Under long-term moderate drought conditions, the overexpression of *MdATG8i* improved photosynthetic efficiency by contributing to maintaining an optimal stomatal aperture, stimulating ROS scavenging, and protecting the photosynthetic apparatus. The higher accumulation of carbohydrates and amino acids in the OE lines than in the WT plants contributed to mitigating osmotic pressure in the OE lines. Furthermore, our results provide new information on the relationship between *MdATG8i-*mediated autophagy and WUE regulation. The *MdATG8i* overexpression lines provide an optimal material for the future development of apple varieties that exhibit improved WUE with minor growth penalties under long-term moderate drought conditions.

## Materials and methods

### Plant materials and stress treatment

Tissue-cultured GL-3 plantlets of *M. domestica* (Royal Gala) and *MdATG8i-*OE lines were cultured as described previously^37^. The plants were cultivated on MS agar medium containing 0.2 mg L^−1^ IAA and 0.2 mg L^−1^ 6BA for 30 days. Then, after rooting on MS agar medium containing 0.5 mg L^−1^ IAA and 0.5 mg L^−1^ IBA for 40 days, the WT and transgenic seedlings were moved to black plastic pots containing a mixture of soil/roseite/perlite (v:v:v, 3:1:1). After culturing for 30 days in the pots, the seedlings were transferred to larger pots (38 × 23 cm) containing equal mixtures of soil/sand/organic matter (v:v:v, 5:1:1) and were grown in a semiopen greenhouse at Northwest A&F University Yangling (34°20’N, 108°24’E), Shaanxi Province, China under environmental conditions that were similar to field conditions.

Two-year-old plants of similar size were assigned to two treatment groups: (i) normal conditions, daily irrigation to maintain 75–85% field capacity, and (ii) moderate drought conditions, daily irrigation to maintain 45–55% field capacity. On day 80 of this experiment, the 8th to 11th leaves from the base of the stem were collected from 15 plants per treatment. The leaves were rapidly frozen in liquid nitrogen and stored at −80 °C.

### RNA extraction and quantitative real-time polymerase chain reaction (PCR) analysis

Total RNA was extracted as previously described by Huo et al.^37^. Quantitative real-time PCR was carried out following Sun et al.^45^. The primer sequences used in the expression analysis are listed in Table S1.

### Determinations of the growth parameters and WUE_L_

The measurements of plant height and stem diameter were performed on days 0 and 80 of the long-term drought treatment. Plant heights were measured with a flexible ruler. The stem diameter was determined at 10 cm above the stem base using a digital micrometer (0.001 mm). On days 0 and 80 of this experiment, ten plants in each treatment were harvested. After the TFW was calculated, the samples were dried in an oven at 65 °C for 2 weeks. Subsequently, the TDW was calculated. The RGR was computed as described previously^74^. The WUE_L_ was calculated using the following formula:

$${\mathrm{WUE}}_{\mathrm{L}} = \left( {{\mathrm{TDW}}2 - {\mathrm{TDW}}1} \right)/W$$

where TDW2 is the TDW of the plant on day 80, TDW1 is the TDW of the plant on day 0, and *W* is the water consumed during the treatment period^75^.

### Determinations of Pn and chlorophyll fluorescence parameters

Photosynthetic parameters were determined on sunny days (09:00 to 11:00 hours) using a LI-COR 6400 portable photosynthesis system (Li-COR, Huntington Beach, CA, USA). The measurements were performed following Sun et al.^31^.

Chlorophyll fluorescence was monitored with a Dual-PAM-100 system (Heinz Walz, Effeltrich, Germany), as reported previously^76^. The chlorophyll fluorescence parameters were measured after the leaves were dark-adapted for 20 min.

### Determination of physiological indices

The RWC and relative electrolyte leakage in the leaves were measured as reported previously^77,78^. The concentration of chlorophyll was obtained based on the method of Lichtenthaler and Wellburn^79^. The MDA levels of the leaves were measured using the thiobarbituric acid reaction, as previously described by Heath and Packer^80^.

### Determination of ROS accumulation and antioxidant enzyme activity

The accumulation of H_2_O_2_ and O_2_^−^ were measured by staining the leaves in fresh solutions of DAB and NBT, respectively. The activities of SOD and POD and the concentrations of H_2_O_2_ and O_2_^−^ were measured as previously described by Wang et al.^56^.

### Detection of ROS fluorescence in guard cells

The measurements of the ROS level in the guard cells were performed with H_2_DCFDA. The epidermal strips were transferred to 10 mL of loading buffer (10 mM Tris, 50 mM KCl, pH 6.5) and treated with 50 µM H_2_DCFDA in the dark for 30–60 min. Afterward, the epidermal strips were washed with loading buffer to remove the excess dye. The fluorescence was determined using confocal microscopy (TCS SP8 SR; Leica) with an excitation wavelength of 460–500 nm.

### Determinations of starch, soluble sugars, and amino acids

The starch measurements were performed as previously described by Sun et al.^50^. The extractions and derivations of the soluble sugars were performed as previously described by Hu et al.^81^. The extractions of the amino acids were performed as previously described, with minor modifications^37^. Briefly, 0.1 g of the leaf samples was extracted with 1 mL of 50% ethanol mixed with 0.1 mol/L HCl and centrifuged at 12,000 × *g* for 10 min. The supernatants were then filtered through 0.22-μm organic filters and diluted 20 times with methanol to measure the concentrations of metabolites using high-performance liquid chromatography–mass spectrometry (QTRAP5500; AB, America).

### Observations of the leaf stomata, chloroplasts, and autophagic bodies

After 80 days of the long-term moderate drought treatment, the leaves at the fourth position from the top of the plants were sampled. These samples were immediately cut into small pieces and were fixed in a 4% glutaraldehyde solution in 0.1 M phosphate-buffered saline (pH 6.8) before being maintained at 4 °C for 12 h. Observations of the leaf stomata were performed with a scanning electron microscope (JSM-6360LV; JEOL Ltd., Tokyo, Japan), as previously described by Liang et al.^55^. Observations of the chloroplast and autophagic bodies were conducted using a transmission electron microscope (JEOL-1230; Hitachi, Tokyo, Japan), as previously reported by Sun et al.^31^. The stomatal density and aperture were measured with the ImageJ software.

### Statistical analysis

SPSS 22.0 software was used for statistical data analysis. All experimental data were subjected to one-way analysis of variance, and the differences between means were assessed by Tukey’s multiple range tests (*P* < 0.05). The values are represented as means ± standard deviations.

## Supplementary information

Supplemental material

## References

[1] Chaitanya KV, Jutur PP, Sundar D, Reddy AR (2003). Water stress effects on photosynthesis in different mulberry cultivars. Plant Growth Regul..

[2] Bhatt RM, Rao N (2005). Influence of pod load on response of okra to water stress. Indian J. Plant Physiol..

[3] Zhang N, Zhao B, Zhang HJ, Weeda S, Guo YD (2012). Melatonin promotes water-stress tolerance, lateral root formation, and seed germination in cucumber (*Cucumis sativus* L.). J. Pineal Res..

[4] Jury W, Vaux H (2005). The role of science in solving the world’s emerging water problems. Proc. Natl Acad. Sci. USA.

[5] Shao HB, Chu LY, Jaleel CA, Zhao CX (2008). Water-deficit stress-induced anatomical changes in higher plants. C R Biol..

[6] Condon AG, Richards R, Rebetzke G, Farquhar G (2004). Breeding for high water-use efficiency. J. Exp. Bot..

[7] Yamori W (2016). Enhanced leaf photosynthesis as a target to increase grain yield: insights from transgenic rice lines with variable Rieske FeS protein content in the cytochrome b (6)/f complex. Plant Cell Environ..

[8] Stitt M, Lunn J, Usadel B (2010). *Arabidopsis* and primary photosynthetic metabolism - more than the icing on the cake. Plant J..

[9] Simkin A, Lopez-Calcagno P, Raines C (2019). Feeding the world: improving photosynthetic efficiency for sustainable crop production. J. Exp. Bot..

[10] Condon AG (2020). Drying times: plant traits to improve crop water use efficiency and yield. J. Exp. Bot..

[11] Gururani MA, Venkatesh J, Tran LS (2015). Regulation of photosynthesis during abiotic stress-induced photoinhibition. Mol. Plant.

[12] Joshi R, Karan R, Singla-Pareek SL, Pareek A (2016). Ectopic expression of pokkali phosphoglycerate kinase-2 (ospgk2-p) improves yield in tobacco plants under salinity stress. Plant Cell Rep..

[13] Maxwell K, Johnson GN (2000). Chlorophyll fluorescence–a practical guide. J. Exp. Bot..

[14] Hare PD, Cress WA, Van StadenJ (2000). Dissecting the roles of osmolyte accumulation during stress. Plant Cell Environ..

[15] Rohit J (2019). Enhancing trehalose biosynthesis improves yield potential in marker-free transgenic rice under drought, saline, and sodic conditions. J. Exp. Bot..

[16] Shu L (2011). Genetic, proteomic and metabolic analysis of the regulation of energy storage in rice seedlings in response to drought. Proteomics.

[17] Zanella M (2016). Î²-amylase 1 (BAM1) degrades transitory starch to sustain proline biosynthesis during drought stress. J. Exp. Bot..

[18] Rosa M (2009). Soluble sugars–metabolism, sensing and abiotic stress: a complex network in the life of plants. Plant Signal. Behav..

[19] Ruan YL, Jin Y, Yang YJ, Li GJ, Boyer JS (2010). Sugar input, metabolism, and signaling mediated by invertase: roles in development, yield potential, and response to drought and heat. Mol. Plant.

[20] Sala A, Woodruff DR, Meinzer FC (2012). Carbon dynamics in trees: feast or famine?. Tree Physiol..

[21] Wei T (2019). Enhanced ROS scavenging and sugar accumulation contribute to drought tolerance of naturally occurring autotetraploids in *Poncirus trifoliata*. Plant Biotechnol. J..

[22] Bowne JB (2012). Drought responses of leaf tissues from wheat cultivars of differing drought tolerance at the metabolite level. Mol. Plant..

[23] Tan Z, Wen X, Wang Y (2020). Betula platyphylla *BpHOX2* transcription factor binds to different cis‐acting elements and confers osmotic tolerance. J. Integr. Plant Biol..

[24] Szabados L, Savouré A (2010). Proline: a multifunctional amino acid. Trends Plant Sci..

[25] Izumi M, Hidema J, Makino A, Ishida H (2013). Autophagy contributes to nighttime energy availability for growth in. Arabidopsis. Plant Physiol..

[26] Bassham DC (2006). Autophagy in development and stress responses of plants. Autophagy.

[27] Marshall RS, Vierstra RD (2018). Autophagy: the master of bulk and selective recycling. Annu. Rev. Plant Biol..

[28] Mizushima N, Yoshimori T, Ohsumi Y (2011). The role of Atg proteins in autophagosome formation. Annu. Rev. Cell Dev. Biol..

[29] Mizushima N (1998). A protein conjugation system essential for autophagy. Nature.

[30] Luo L (2017). Autophagy is rapidly induced by salt stress and is required for salt tolerance in *Arabidopsis*. Front. Plant Sci..

[31] Sun X (2018). Improvement of drought tolerance by overexpressing *MdATG18a* is mediated by modified antioxidant system and activated autophagy in transgenic apple. Plant Biotechnol. J..

[32] Liu Y, Xiong Y, Bassham DC (2009). Autophagy is required for tolerance of drought and salt stress in plants. Autophagy.

[33] Wang P, Sun X, Jia X, Ma F (2017). Apple autophagy-related protein *MdATG3s* afford tolerance to multiple abiotic stresses. Plant Sci..

[34] Wang Y, Cai S, Yin L, Shi K, Zhou J (2015). Tomato *HsfA1a* plays a critical role in plant drought tolerance by activating ATG genes and inducing autophagy. Autophagy.

[35] NBSC. *National Database* (National Bureau of Statistics of China, 2016).

[36] Wang P (2016). Characterization of an autophagy-related gene *MdATG8i* from apple. Front. Plant Sci..

[37] Huo L (2020). *MdATG8i* functions positively in apple salt tolerance by maintaining photosynthetic ability and increasing the accumulation of arginine and polyamines. Environ. Exp. Bot..

[38] Zhou S (2015). Physiological and proteome analysis suggest critical roles for the photosynthetic system for high water-use efficiency under drought stress in Malus. Plant Sci..

[39] Gómez-Bellot M (2013). Water relations, nutrient content and developmental responses of Euonymus plants irrigated with water of different degrees of salinity and quality. J. Plant Res..

[40] Lee JS (2012). Direct interaction of ligand-receptor pairs specifying stomatal patterning. Genes Dev..

[41] Hara K, Kajita R, Torii KU, Bergmann DC, Kakimoto T (2007). The secretory peptide gene EPF1 enforces the stomatal one-cell-spacing rule. Genes Dev..

[42] Jiang Q (2019). Overexpression of *MdEPF2* improves water use efficiency and reduces oxidative stress in tomato. Environ. Exp. Bot..

[43] Kondo T (2010). Stomatal density is controlled by a mesophyll-derived signaling molecule. Plant Cell Physiol..

[44] Zhu Y (2016). The regulatory role of silicon on carbohydrate metabolism in *Cucumis sativus* L. under salt stress. Plant Soil.

[45] Zandalinas SI, Mittler R, Balfagón D, Arbona V, Gómez-Cadenas A (2018). Plant adaptations to the combination of drought and high temperatures. Physiol. Plant.

[46] Bertolino LT, Caine RS, Gray JE (2019). Impact of stomatal density and morphology on water-use efficiency in a Changing world. Front. Plant Sci..

[47] Avin-Wittenberg T (2019). Autophagy and its role in plant abiotic stress management. Plant Cell Environ..

[48] Huo L, Guo Z, Zhang Z, Jia X, Ma F (2020). The apple autophagy-related gene *MdATG9* confers tolerance to low nitrogen in transgenic apple callus. Front. Plant Sci..

[49] Huo L (2020). *MdATG18a* overexpression improves basal thermotolerance in transgenic apple by decreasing damage to chloroplasts. Hortic. Res..

[50] Sun X (2017). *MdATG18a* overexpression improves tolerance to nitrogen deficiency and regulates anthocyanin accumulation through increased autophagy in transgenic apple. Plant Cell Environ..

[51] Long SP, Zhu XG, Naidu SL, Ort DR (2006). Can improvement in photosynthesis increase crop yields?. Plant Cell Environ..

[52] McLoughlin F (2018). Maize multi-omics reveal roles for autophagic recycling in proteome remodelling and lipid turnover. Nat. Plants.

[53] Marien Havé (2019). Proteomic and lipidomic analyses of the *Arabidopsis* atg5 autophagy mutant reveal major changes in endoplasmic reticulum and peroxisome metabolisms and in lipid composition. N. Phytol..

[54] Chaves MM, Flexas J, Pinheiro C (2008). Photosynthesis under drought and salt stress: regulation mechanisms from whole plant to cell. Ann. Bot..

[55] Liang B (2018). Effects of exogenous dopamine on the uptake, transport, and resorption of apple ionome under moderate drought. Front. Plant Sci..

[56] Wang Q (2019). High-efficient utilization and uptake of N contribute to higher NUE of ‘Qinguan’ apple under drought and N-deficient conditions compared with ‘Honeycrisp’. Tree Physiol..

[57] Liu Y (2017). Trithorax-group proteins ARABIDOPSIS TRITHORAX4 (ATX4) and ATX5 function in abscisic acid and dehydration stress responses. N. Phytol..

[58] Yamauchi S, Mano S, Oikawa K, Hikino K, Takemiya A (2019). Autophagy controls reactive oxygen species homeostasis in guard cells that is essential for stomatal opening. Proc. Natl Acad. Sci. USA.

[59] Havaux M (1993). Characterization of thermal damage to the photosynthetic electron transport system in potato leaves. Plant Sci..

[60] Izumi M, Ishida H, Nakamura S, Hidema J (2017). Entire photodamaged chloroplasts are transported to the central vacuole by autophagy. Plant Cell.

[61] Krasensky J, Jonak C (2012). Drought, salt, and temperature stress-induced metabolic rearrangements and regulatory networks. J. Exp. Bot..

[62] Gong X, Liu M, Zhang L, Ruan Y, Wang C (2014). *Arabidopsis AtSUC2* and *AtSUC4*, encoding sucrose transporters, are required for abiotic stress tolerance in an ABA-dependent pathway. Physiol. Plant.

[63] Hosseini SA, Hajirezaei MR, Seiler C, Sreenivasulu N, von, Wirén N (2016). A potential role of flag leaf potassium in conferring tolerance to drought-induced leaf senescence in barley. Front. Plant Sci..

[64] Rui G (2018). Metabolic responses to drought stress in the tissues of drought-tolerant and drought-sensitive wheat genotype seedlings. AoB Plants.

[65] Blum A (2017). Osmotic adjustment is a prime drought stress adaptive engine in support of plant production. Plant Cell Environ..

[66] Guiboileau A (2013). Physiological and metabolic consequences of autophagy deficiency for the management of nitrogen and protein resources in *Arabidopsis* leaves depending on nitrate availability. N. Phytol..

[67] Masclaux-Daubresse C (2014). Stitching together the multiple dimensions of autophagy using metabolomics and transcriptomics reveals impacts on metabolism, development, and plant responses to the environment in *Arabidopsis*. Plant Cell.

[68] Mizushima N (2007). Autophagy: process and function. Genes Dev..

[69] Wang Y (2013). Autophagy contributes to leaf starch degradation. Plant Cell.

[70] Du Y (2019). Effect of drought stress on sugar metabolism in leaves and roots of soybean seedlings. Plant Physiol. Biochem..

[71] Huot B, Yao J, Montgomery B, He S (2014). Growth–defense tradeoffs in plants: a balancing act to optimize fitness. Mol. Plant.

[72] Bozhkov PV (2018). Plant autophagy: mechanisms and functions. J. Exp. Bot..

[73] Minina EA (2018). Transcriptional stimulation of rate-limiting components of the autophagic pathway improves plant fitness. J. Exp. Bot..

[74] Radford JP (1967). Growth analysis formulae - their use and abuse. Crop Sci..

[75] Ehdaie B (1995). Variation in water-use efficiency and its components in wheat: II. pot and field experiments. Crop Sci..

[76] Deng C, Zhang D, Pan X, Chang F, Wang S (2013). Toxic effects of mercury on PSI and PSII activities, membrane potential and transthylakoid proton gradient in *Microsorium pteropus*. J. Photochem. Photobiol. B.

[77] Dionisio-Sese ML, Tobita S (1998). Antioxidant responses of rice seedlings to salinity stress. Plant Sci..

[78] Gaxiola RA (2001). Drought- and salt-tolerant plants result from overexpression of the AVP1 H^+^-pump. Proc. Natl Acad. Sci. USA.

[79] Lichtenthaler HK, Wellburn AR (1983). Determinations of total carotenoids and chlorophylls a and b of leaf extracts in different solvents. Biochem. Soc. Trans..

[80] Heath RL, Packer L (1968). Photoperoxidation in isolated chloroplasts: I. Kinetics and stoichiometry of fatty acid peroxidation. Arch. Biochem. Biophys..

[81] Hu L (2018). Exogenous myo-inositol alleviates salinity-induced stress in *Malus hupehensis**Rehd*. Plant Physiol. Biochem..

