# Supplementary material for: Overexpression of MdATG8i improves water use efficiency in transgenic apple by modulating photosynthesis, osmotic balance, and autophagic activity under moderate water deficit
Source: Hortic Res. 2021 Apr 1;8:81. doi: 10.1038/s41438-021-00521-2 (PMC8012348; doi:10.1038/s41438-021-00521-2)
Supplement: Supplementary file 1 — Supplemental material [file 41438_2021_521_MOESM1_ESM.docx]

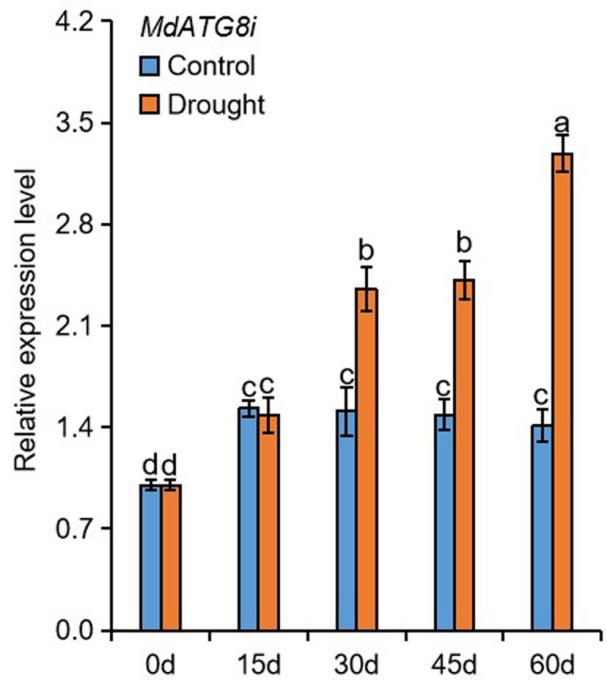


Fig. S1. The relative expression of *MdATG8i* in the GL-3 apple plants exposed to long-term moderate drought stress. The data are the means of three replicates with SD.


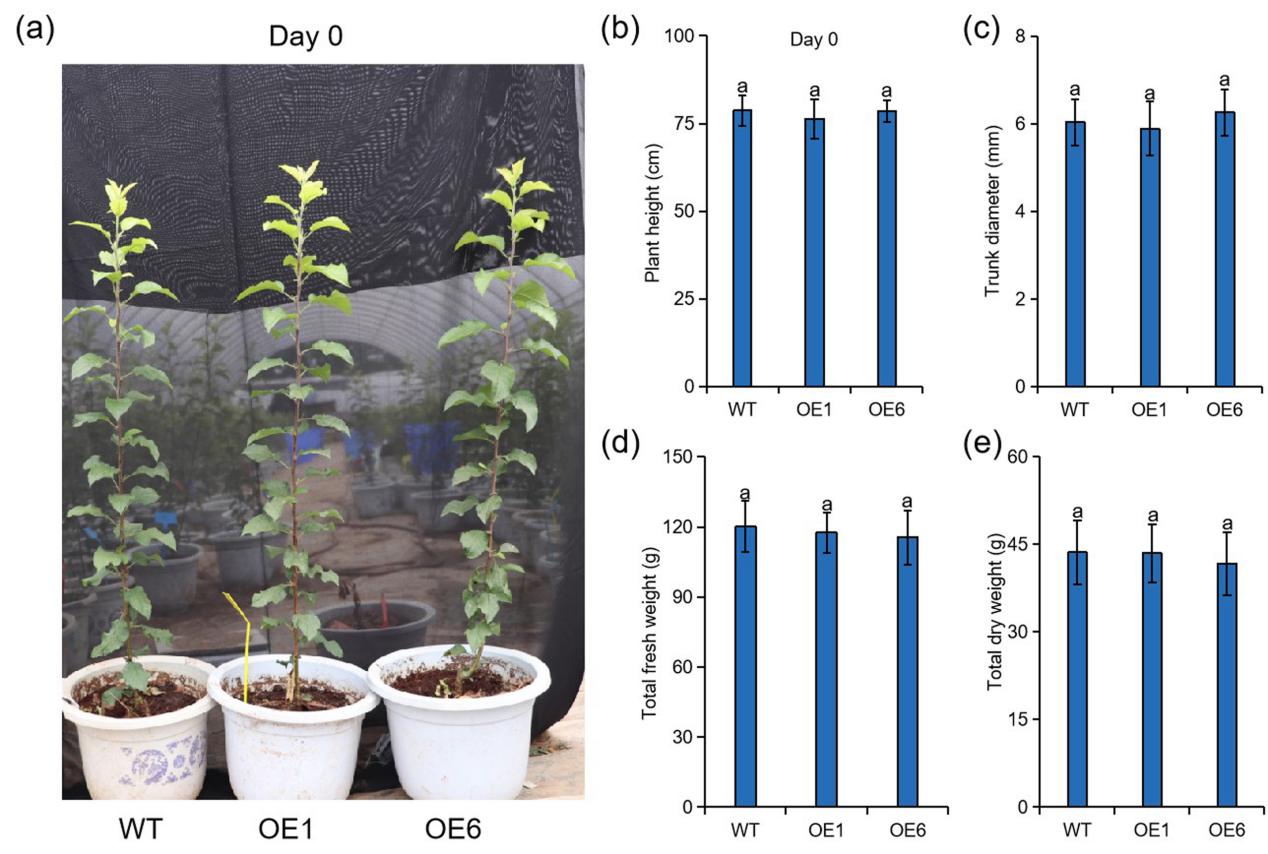


Fig. S2. Growth phenotype of WT and transgenic plants on Day 0. (a) Representative images of WT and transgenic plants. Plant height (b), trunk diameter (c), total fresh weight (d), and total dry weight (e) of WT and transgenic plants were measured on day 0. The data are the means of six replicates with SD. Values not followed by the same letters are significantly different according to one-way ANOVA and Tukey’s multiple range test (P<0.05).


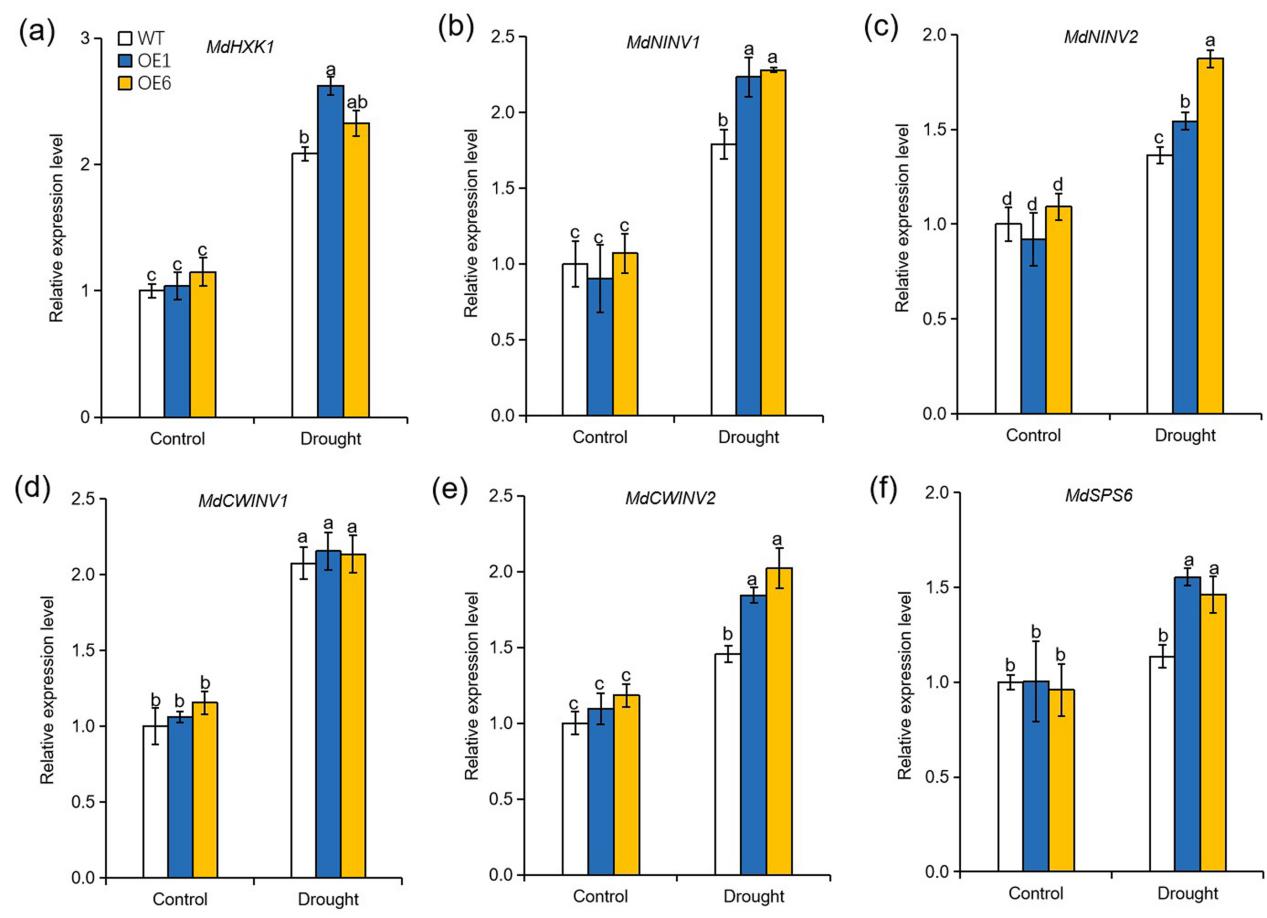


Fig.S3. Gene expression analysis of *MdHXK1* (a), *MdNINV1* (b), *MdNINV2* (c), *MdCWINV1* (d), *MdCWINV2* (e), and *MdSPS6* (f) in WT and transgenic plants after 80 d of cultivation under well-watered or moderate drought conditions. The data are the means of three replicates with SD. Values not followed by the same letters are significantly different according to one-way ANOVA and Tukey’s multiple range test (P<0.05).


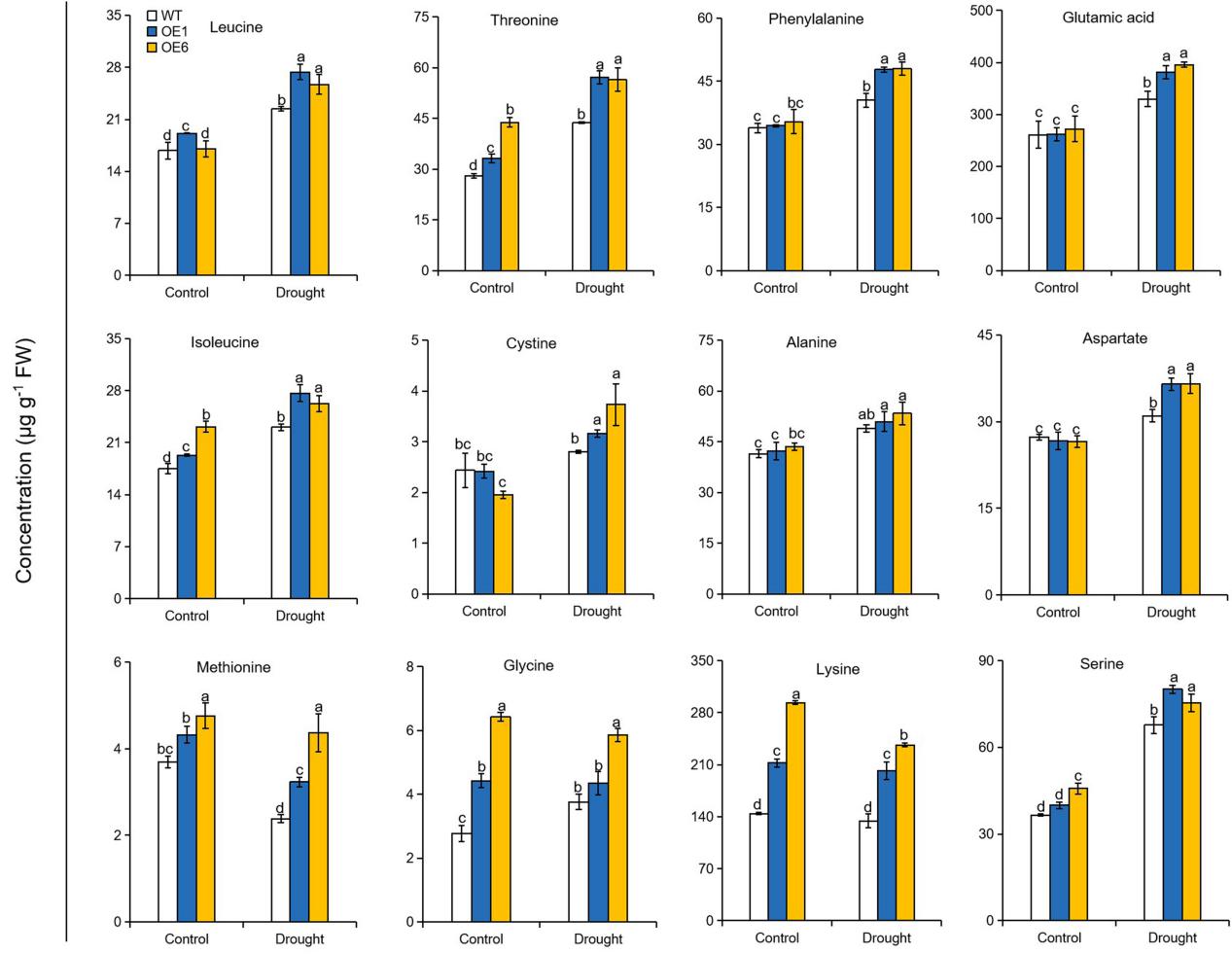


Fig.S4. Amino acids levels in the leaves of WT and transgenic plants subjected to drought as determined using LC–MS. The data are the means of three replicates with SD. Values not followed by the same letters are significantly different according to one-way ANOVA and Tukey’s multiple range test (P<0.05).

Table. S1. Primers used in this study.

| **Name** | **Sequence (5**'**-3**'**)** | **Purpose** |
| --- | --- | --- |
| *MDH* | F: CGTGATTGGGTACTTGGAAC | Reference gene used in real-time PCR |
|  | R:TGGCAAGTGACTGGGAATGA |  |
| q*ATG8i* | F: GCAGCAGGCTTCACTTGACTCC | Quantitative expression of *MdATG8i* |
|  | R: GGAATCCATGCGACTGGCTGTT |  |
| q*ATG3a* | F: AAGGGGGCGGAGATGGTTC | Quantitative expression of *MdATG3a* |
|  | R: GCACTTAGAGACGAGGTTATCGC |  |
| *qATG3b* | F: AGGGAGATGGTTTTGAAACAGA | Quantitative expression of *MdATG3b* |
|  | R: ACTTAGAGACGAGGTTATCGC |  |
| qATG4a | F: GCCTCCAAGCTGGCAGATGAATC | Quantitative expression of *MdATG4a* |
|  | R: CCACTATCACCCAACGCATCACTG |  |
| qATG5a | F: GCAGGTCGTGTTCCAGTTC | Quantitative expression of *MdATG5a* |
|  | R: CCTCCTCCTCCTTGTATCTCAA |  |
| q*ATG7a* | F: GCGGATATGAGCAACCTTGGC | Quantitative expression of *MdATG7a* |
|  | R: ATCAATAGGCGCAACGACATCA |  |
| q*ATG7b* | F: ATCGGTAACAGGAGTAAGTCGG | Quantitative expression of *MdATG7b* |
|  | R: TTTATCAAGCGCATGAAAGCCT |  |
| q*ATG9* | F: ACTTCATGCGTCAGCCTTCAGA | Quantitative expression of *MdATG9* |
|  | R: CGTTCCTCCAATCCAACCGTTG |  |
| q*ATG10* | F: TGGAACCAGCGAGTGGATGAAG | Quantitative expression of *MdATG10* |
|  | R: ACAACTGAGAGCCAAGACACCA |  |
| *qATG12* | F: ACAGTGCATTCTCGCCAAACCC | Quantitative expression of *MdATG12* |
|  | R: CCCCATGCCATGGAGCAAGC |  |
| q*ATG18a* | F: ATGATTCCAGGCTTGCCTGCTTTG | Quantitative expression of *MdATG18a* |
|  | R: TGCAGCAAAGTTCCGTCGAGAGTA |  |
| *qMdNCED3* | F: GCAGGAGATGATCGGCG | Quantitative expression of *MdNCED3* |
|  | R: CAGAAGCAGTCGGGGCAGT |  |
| *qMdABI1* | F: GGGAGGAACAACAAGGGA | Quantitative expression of *MdABI1* |
|  | R: AAGAAATGAACGGGTGAGAT |  |
| *q**MdABI2* | F: GACGACGAATGCCTAATT | Quantitative expression of *MdABI2* |
|  | R: TCTTGTGCCAGAGGAGTA |  |
| *qMdEPF1* | F: CGCATAGCTCGGCCTCATT | Quantitative expression of *MdEPF1* |
|  | R: CGACCCTGCCACTTGGACT |  |
| *qMdEPF2* | F: TACGTGTTTGGCCTCCTCA | Quantitative expression of *MdEPF2* |
|  | R: CCTCGATCCGCTTTGGTAT |  |
| *qMdEPFL5* | F: AGCCTCAACGGCAGTAACC | Quantitative expression of *MdEPFL5* |
|  | R: GCGATGAACCCAGACGAAT |  |
| *qMdEPFL9* | F: ACAAGGGTCCAGAACTCAA | Quantitative expression of *MdEPFL9* |
|  | R: CCTCCACAGGGACTTGCTC |  |
| q*MdHXK1* | F:AACTTCCCTGTTCTGCGTGTAC | Quantitative expression of *MdHXK1* |
|  | R: CAAACCTTCTCCTTCCGTAGC |  |
| *qMdNINV1* | F: GTCCATTGTTTCATCATTGGGTAC | Quantitative expression of *MdNINV1* |
|  | R: GGTCGCTGCCAGTGATTATACG |  |
| *qMdNINV2* | F: GAGTTCCAGACAGGCATAAGGCT | Quantitative expression of *MdNINV2* |
|  | R: CCATCCGTCTATCAATCATACAGG |  |
| *qMdCWINV1* | F: TAACAAATATGTGGTGCTCCTCTG | Quantitative expression of *MdCWINV1* |
|  | R: ACCCTAGCTGTTATGCACGCCT |  |
| *qMdCWINV2* | F: TTCAAAGCTAAAGGCAGACACG | Quantitative expression of *MdCWINV2* |
|  | R: GTAAATCTACATCTACAAAGCCAGC |  |
| *qMdSPS6* | F: AGGTTCTGTTGAGTATGGCAGTGAG | Quantitative expression of *MdSPS6* |
|  | R: GTGCTTCAAGTGCCGCTGAGA |  |
